# Mycobacteriophage Tarkin: a Cluster E Phage

**DOI:** 10.1128/mra.00961-22

**Published:** 2022-11-21

**Authors:** Katherine E. Cleary, Anna M. Fakhri, Ethan N. Dionne, Marcie Warner, Joseph A. DeGiorgis, Kathleen Cornely

**Affiliations:** a Department of Chemistry and Biochemistry, Providence College, Providence, Rhode Island, USA; b Department of Biological Sciences, University of Pittsburgh, Pittsburgh, Pennsylvania, USA; c Department of Biology, Providence College, Providence, Rhode Island, USA; d Whitman Center, Marine Biological Laboratory, Woods Hole, Massachusetts, USA; Queens College CUNY

## Abstract

Mycobacteriophage Tarkin is a newly isolated phage that infects Mycobacterium smegmatis mc^2^155. Tarkin was discovered in Providence, RI, and has a 75,998-bp genome sequence. Tarkin is predicted to have 142 protein coding genes and 2 tRNA genes. Based on gene content similarity, Tarkin is grouped with mycobacteriophages in cluster E.

## ANNOUNCEMENT

Mycobacteriophage Tarkin was discovered through our participation in the Science Education Alliance—Phage Hunters Advancing Genomics and Evolutionary Science (SEA-PHAGES) program (1). To date, more than 2,000 mycobacteriophages have been isolated and sequenced; some of these also infect pathogenic mycobacteria. With the increasing occurrence of antibiotic-resistant pathogens constituting a global threat to public health, phage therapy has reemerged as a viable treatment strategy. Recently, cocktails of mycobacteriophages, some of them discovered by participants in the SEA-PHAGES program, were successfully used to treat patients suffering from antibiotic-resistant strains of Mycobacterium abscessus (2) and Mycobacterium chelonae (3).

**FIG 1:**
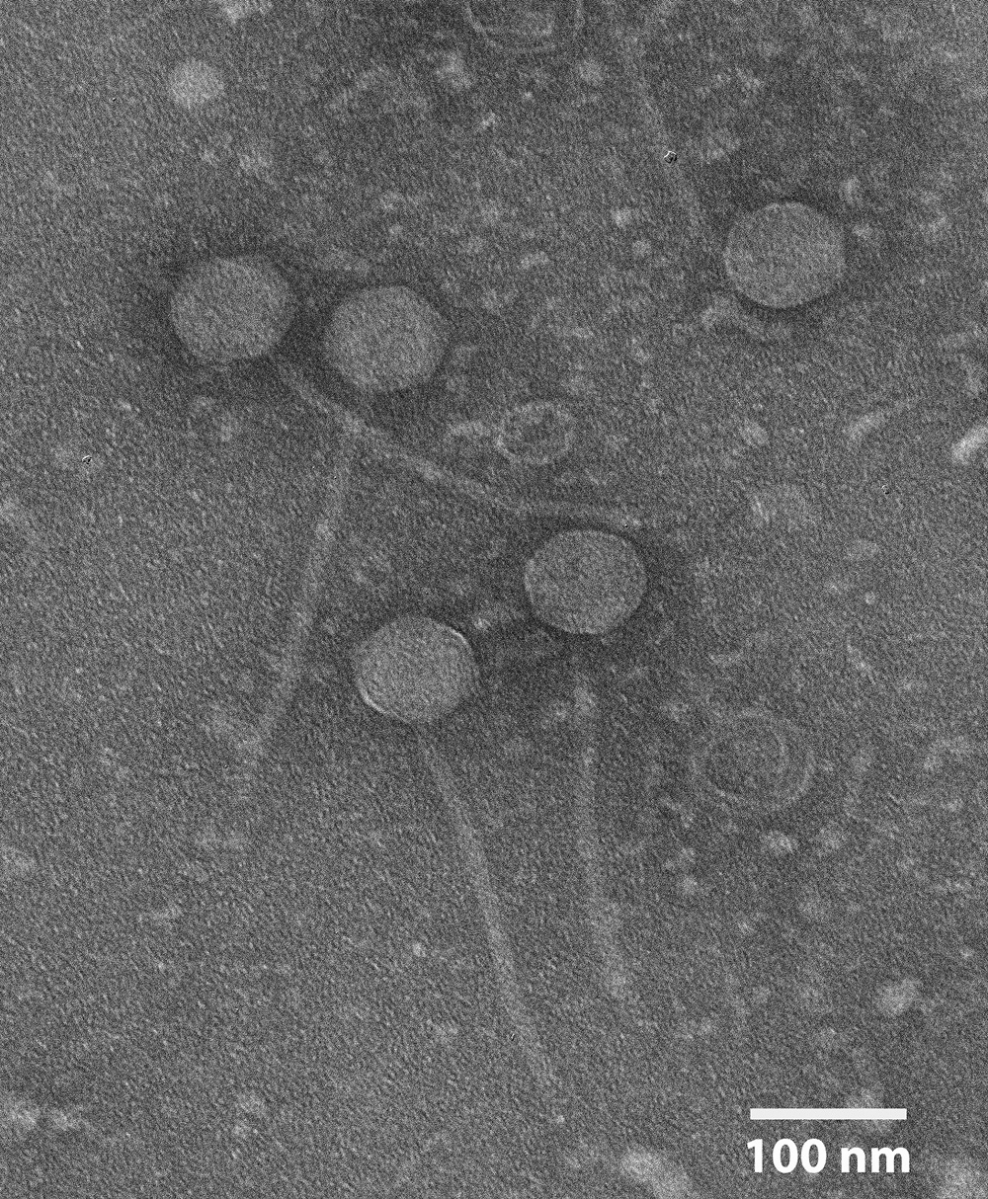
Image of Tarkin created using a JEOL 200CX transmission electron microscope. Electron microscopy revealed that Tarkin is a member of the *Siphoviridae* family, with a circular capsid of 75 to 85 nm and a straight tail 235 to 275 nm long.

Mycobacteriophage Tarkin was isolated from a peaty surface soil sample collected in Providence, RI (Table 1), using standard methods (4). The soil sample was washed in 7H9 liquid medium, the wash filtered (pore size, 0.02 μm), and the filtrate inoculated with Mycobacterium smegmatis mc^2^155. After 2 days of shaking at 37°C, the filtrate was examined for phages by plating in top agar with M. smegmatis, yielding phage Tarkin. Tarkin, which was purified through five rounds of plating, formed clear plaques ~2 mm in diameter after an overnight incubation at 37°C. Negative-stain transmission electron microscopy revealed that Tarkin exhibits siphovirus morphology (Fig. 1). Phage DNA was isolated from high-titer lysates using phenol/chloroform/isoamyl alcohol (25:24:1) (5) and sequenced at the University of Pittsburgh (Table 1). The reads were assembled using Newbler v2.9 (6) and verified using Consed v29.0 (7).

**TABLE 1:** Sequencing, genome, and phage characteristics

Parameter	Phage data
Sequencing	
Sequencing instrument	Illumina MiSeq (v3 reagent)
Library prep kit	NEB Ultra II
No. of reads	490,299
Length of reads (bp)	150 (single-end reads)
Shotgun coverage (×)	911
Phage characteristics	
Genome length (bp)	75,998
3′ single-stranded overhang	9 bases (5′-CGCTTGTCA-3′)
GC content (%)	63.0
attP site (bp)	40362–40385
Sample characteristics	
Collection date	5 September 2018
Collection location coordinates	41.843 N, 71.438 W
Capsid size (nm)	73–79
No. of particles measured	5
Mean (nm)	75
Tail length (nm)	235–275
No. of particles measured	5
Mean (nm)	253

The Tarkin genome is 75,998 bp long and has 63.0% GC content (Table 1). Based on its gene content similarity of ~35% to phages in the Actinobacteriophage Database (https://phagesdb.org/) using BLASTn v2.2.26 (8), Tarkin was assigned to cluster E (9–12). DNA Master v5.22.2 was used to perform the annotation (http://cobamide2.bio.pitt.edu/) (13), with GeneMark v2.5 (14), Gimmer v3.02 (15), and Starterator v1.1 (http://phages.wustl.edu/starterator/) (16) used to call the start sites. Two tRNAs were identified using tRNAscan-SE (17) and Aragorn (18). Protein coding gene functions were determined using BLASTp v2.9 (8), TMHMM v2.0 (19), HHpred (PDB, Pfam, UniProt databases) (20), and the NCBI Conserved Domain Database (21) and by comparing Tarkin’s Phamerator (22) map to that of similar phages. Functions were assigned to 46 of the 142 genes. All tools were run with default parameters unless specified otherwise. Tarkin possesses immunity repressor (gp54) and integrase (gp51) genes, indicating the potential for a temperate lifestyle.

Mycobacteriophage Tarkin is most closely related to the E cluster phages Paperbeatsrock (GenBank accession number MH727557), Kanye (MN428059), and ShereKhan (MH513983), determined using Phamerator (22). We noted that the DNA methylase (gp98) in Tarkin is 1,739 bp, over 500 bp longer than the homolog in similar E cluster phages. This adenine- and cytosine-specific methylase could defend against host restriction systems similarly to phage CrystalP (KY319168) (23). In addition, we determined that the attP site is located within the coding region of Tarkin’s repressor protein. Such positioning has been shown to serve as a recombination-based switch for establishing lysogeny (24). A similar attP site has been located in E cluster phage Ukulele (KT373978) (25).

### Data availability.

The GenBank accession number for the Tarkin genome sequence is MZ681505. The SRA accession number is SRX14485094.
